# Heterogeneity of Breast Cancer Associations with Five Susceptibility Loci by Clinical and Pathological Characteristics

**DOI:** 10.1371/journal.pgen.1000054

**Published:** 2008-04-25

**Authors:** Montserrat Garcia-Closas, Per Hall, Heli Nevanlinna, Karen Pooley, Jonathan Morrison, Douglas A. Richesson, Stig E. Bojesen, Børge G. Nordestgaard, Christen K. Axelsson, Jose I. Arias, Roger L. Milne, Gloria Ribas, Anna González-Neira, Javier Benítez, Pilar Zamora, Hiltrud Brauch, Christina Justenhoven, Ute Hamann, Yon-Dschun Ko, Thomas Bruening, Susanne Haas, Thilo Dörk, Peter Schürmann, Peter Hillemanns, Natalia Bogdanova, Michael Bremer, Johann Hinrich Karstens, Rainer Fagerholm, Kirsimari Aaltonen, Kristiina Aittomäki, Karl von Smitten, Carl Blomqvist, Arto Mannermaa, Matti Uusitupa, Matti Eskelinen, Maria Tengström, Veli-Matti Kosma, Vesa Kataja, Georgia Chenevix-Trench, Amanda B. Spurdle, Jonathan Beesley, Xiaoqing Chen, Australian Ovarian Cancer Management Group, The Kathleen Cuningham Foundation Consortium for Research into Familial Breast Cancer, Peter Devilee, Christi J. van Asperen, Catharina E. Jacobi, Rob A. E. M. Tollenaar, Petra E.A. Huijts, Jan G. M. Klijn, Jenny Chang-Claude, Silke Kropp, Tracy Slanger, Dieter Flesch-Janys, Elke Mutschelknauss, Ramona Salazar, Shan Wang-Gohrke, Fergus Couch, Ellen L. Goode, Janet E. Olson, Celine Vachon, Zachary S. Fredericksen, Graham G. Giles, Laura Baglietto, Gianluca Severi, John L. Hopper, Dallas R. English, Melissa C. Southey, Christopher A. Haiman, Brian E. Henderson, Laurence N. Kolonel, Loic Le Marchand, Daniel O. Stram, David J. Hunter, Susan E. Hankinson, David G. Cox, Rulla Tamimi, Peter Kraft, Mark E. Sherman, Stephen J. Chanock, Jolanta Lissowska, Louise A. Brinton, Beata Peplonska, Jan G. M. Klijn, Maartje J. Hooning, Han Meijers-Heijboer, J. Margriet Collee, Ans van den Ouweland, Andre G. Uitterlinden, Jianjun Liu, Low Yen Lin, Li Yuqing, Keith Humphreys, Kamila Czene, Angela Cox, Sabapathy P. Balasubramanian, Simon S. Cross, Malcolm W. R. Reed, Fiona Blows, Kristy Driver, Alison Dunning, Jonathan Tyrer, Bruce A. J. Ponder, Suleeporn Sangrajrang, Paul Brennan, James McKay, Fabrice Odefrey, Valerie Gabrieau, Alice Sigurdson, Michele Doody, Jeffrey P. Struewing, Bruce Alexander, Douglas F. Easton, Paul D. Pharoah

**Affiliations:** 1 Division of Cancer Epidemiology and Genetics, National Cancer Institute, Rockville, Marylan, United States of America; 2 Department of Medical Epidemiology and Biostatistics, Karolinska Institute, Stockholm, Sweden; 3 Department of Obstetrics and Gynaecology, Helsinki University Central Hospital, Helsinki, Finland; 4 Department of Oncology, University of Cambridge, Cambridge, United Kingdom; 5 Department of Clinical Biochemistry, Herlev and Bispebjerg University Hospitals, University of Copenhagen, Denmark; 6 Department of Bispebjerg University Hospitals, University of Copenhagen, Denmark; 7 Department of Breast Surgery, Herlev University Hospital, University of Copenhagen, Denmark; 8 Spanish National Cancer Centre, Madrid, Spain; 9 Monte Naranco Hospital, Oviedo, Spain; 10 La Paz Hospital, Madrid, Spain; 11 Dr. Margarete Fischer-Bosch Institute of Clinical Pharmacology, Stuttgart and University of Tübingen, Tübingen, Germany; 12 Deutsches Krebsforschungszentrum Heidelberg, Heidelberg, Germany; 13 Evangelische Kliniken Bonn gGmhH Johanniter Krankenhaus, Bonn, Germany; 14 Berufsgenossenschaftliches Forschungsinstitut für Arbeitsmedizin, Ruhr University Bochum, Germany; 15 Institute für Pathology, University Bonn, Bonn, Germany; 16 Department of Gynecology and Obstetrics, Hannover Medical School, Hannover, Germany; 17 Department of Radiation Oncology, Hannover Medical School, Hannover, Germany; 18 Department of Oncology, Helsinki University Central Hospital, Helsinki, Finland; 19 Department of Clinical Genetics, Helsinki University Central Hospital, Helsinki, Finland; 20 Department of Surgery, Helsinki University Central Hospital, Helsinki, Finland; 21 Institute of Clinical Medicine, Pathology and Forensic Medicine, Biocenter Kuopio, University of Kuopio, Kuopio, Finland; 22 Department of Pathology, Kuopio University Hospital, Kuopio, Finland; 23 Department of Public Health and Clinical Nutrition, Biocenter Kuopio, University of Kuopio, Kuopio, Finland; 24 Department of Surgery, Kuopio University Hospital, Kuopio, Finland; 25 Department of Oncology, Kuopio University Hospital, Kuopio, Finland; 26 Department of Oncology, Vaasa Central Hospital, Vaasa, Finland; 27 The Queensland Institute of Medical Research Post Office, Royal Brisbane Hospital, Herston, Queensland, Australia; 28 Peter MacCallum Cancer Institute, East Melbourne, Victoria, Australia; 29 Departments of Human Genetics and Pathology, Leiden University Medical Center, Leiden, The Netherlands; 30 Department of Clinical Genetics, Leiden University Medical Center, Leiden, The Netherlands; 31 Department of Medical Decision Making, Leiden University Medical Center, Leiden, The Netherlands; 32 Department of Surgery, Leiden University Medical Center, Leiden, The Netherlands; 33 Department of Medical Oncology, Family Cancer Clinic, Erasmus MC-Daniel den Hoed Cancer Center, Rotterdam, The Netherlands; 34 Division of Cancer Epidemiology, German Cancer Research Center, Heidelberg, Germany; 35 Institute for Medical Biometrics and Epidemiology, University Clinic Hamburg-Eppendorf, Hamburg, Germany; 36 Bioglobe GmbH, Hamburg, Germany; 37 Molecular Biology Laboratory, Department of Obstetrics and Gynecology, University of Ulm, Ulm, Germany; 38 Mayo Clinic College of Medicine, Rochester, Minnesota, United States of America; 39 Cancer Epidemiology Centre, The Cancer Council Victoria, Melbourne, Victoria, Australia; 40 Centre for MEGA Epidemiology, The University of Melbourne, Melbourne, Victoria, Australia; 41 Genetic Epidemiology Laboratory, Department of Pathology, The University of Melbourne, Melbourne, Victoria, Australia; 42 Department of Preventive Medicine, Keck School of Medicine, University of Southern California, Los Angeles, California, United States of America; 43 Epidemiology Program, Cancer Research Center of Hawaii, University of Hawaii, Honolulu, Hawaii, United States of America; 44 Department of Preventive Medicine, Keck School of Medicine, University of Southern California, Los Angeles, California, United States of America; 45 Program in Molecular and Genetic Epidemiology, Harvard School of Public Health, Boston, Massachusetts, United States of America; 46 Channing Laboratory, Brigham and Women's Hospital and Harvard Medical School, Boston, Massachusetts, United States of America; 47 Program in Molecular and Genetic Epidemiology, Harvard School of Public Health, Boston, Massachusetts, United States of America; 48 Advanced Technology Center, National Cancer Institute, Gaithersburg, Maryland, United States of America; 49 Department of Cancer Epidemiology and Prevention, Cancer Center and M. Sklodowska-Curie Institute of Oncology, Warsaw, Poland; 50 Nofer Institute of Occupational Medicine, Lodz, Poland; 51 Daniel den Hoed Cancer Center, Erasmus Medical Center, Department of Medical Oncology, Rotterdam, The Netherlands; 52 Department of Clinical Genetics, Erasmus Medical Center, Rotterdam, The Netherlands; 53 Department of Internal Medicine, Erasmus Medical Center, Rotterdam, The Netherlands; 54 Human Genetics, Genome Institute of Singapore, Singapore; 55 Institute for Cancer Studies, Sheffield University Medical School, Sheffield, United Kingdom; 56 Academic Unit of Surgical Oncology, Sheffield University Medical School, Sheffield, United Kingdom; 57 Academic Unit of Pathology, Sheffield University Medical School, Sheffield, United Kingdom; 58 Cancer Research UK, Cambridge Research Institute, Cambridge, United Kingdom; 59 Molecular Epidemiology Unit, National Cancer Institute, Ratchathewi, Bangkok, Thailand; 60 International Agency for Research on Cancer, Lyon, France; 61 Office of Population Genomics, National Human Genome Research Institute, Bethesda, Maryland, United Stated of America; 62 Environmental Health Sciences, University of Minnesota, Minneapolis, Minnesota, United States of America; Baylor College of Medicine, United States of America

## Abstract

A three-stage genome-wide association study recently identified single nucleotide polymorphisms (SNPs) in five loci (fibroblast growth receptor 2 (*FGFR2*), trinucleotide repeat containing 9 (*TNRC9*), mitogen-activated protein kinase 3 K1 (*MAP3K1*), 8q24, and lymphocyte-specific protein 1 (*LSP1*)) associated with breast cancer risk. We investigated whether the associations between these SNPs and breast cancer risk varied by clinically important tumor characteristics in up to 23,039 invasive breast cancer cases and 26,273 controls from 20 studies. We also evaluated their influence on overall survival in 13,527 cases from 13 studies. All participants were of European or Asian origin. rs2981582 in *FGFR2* was more strongly related to ER-positive (per-allele OR (95%CI) = 1.31 (1.27–1.36)) than ER-negative (1.08 (1.03–1.14)) disease (P for heterogeneity = 10^−13^). This SNP was also more strongly related to PR-positive, low grade and node positive tumors (P = 10^−5^, 10^−8^, 0.013, respectively). The association for rs13281615 in 8q24 was stronger for ER-positive, PR-positive, and low grade tumors (P = 0.001, 0.011 and 10^−4^, respectively). The differences in the associations between SNPs in *FGFR2* and 8q24 and risk by ER and grade remained significant after permutation adjustment for multiple comparisons and after adjustment for other tumor characteristics. Three SNPs (rs2981582, rs3803662, and rs889312) showed weak but significant associations with ER-negative disease, the strongest association being for rs3803662 in *TNRC9* (1.14 (1.09–1.21)). rs13281615 in 8q24 was associated with an improvement in survival after diagnosis (per-allele HR = 0.90 (0.83–0.97). The association was attenuated and non-significant after adjusting for known prognostic factors. Our findings show that common genetic variants influence the pathological subtype of breast cancer and provide further support for the hypothesis that ER-positive and ER-negative disease are biologically distinct. Understanding the etiologic heterogeneity of breast cancer may ultimately result in improvements in prevention, early detection, and treatment.

## Author Summary

This report from the Breast Cancer Association Consortium evaluates whether common variants in five recently identified breast cancer susceptibility loci (*FGFR2*, *TNRC9*, *MAP3K1*, 8q24, and *LSP1*) influence the clinical presentation of breast cancer and survival after diagnosis. We studied these susceptibility loci in relation to clinically important tumor characteristics in up to 23,039 invasive breast cancer cases and 26,273 controls of European or Asian origin from 20 studies. The association, with overall survival, was evaluated in 13,527 cases from 13 studies. The most notable findings were that the genetic variants in the fibroblast growth factor receptor 2 (*FGFR2*) gene and the 8q24 region were more strongly related to ER-positive than ER-negative disease, and to low rather than high grade tumors. The loci did not significantly influence survival after accounting for known prognostic factors. Analyses indicated that common genetic variants influence the pathological subtype of breast cancer and provide further support for the hypothesis that ER-positive and ER-negative diseases are biologically distinct tumors. Understanding the etiologic heterogeneity of breast cancer may ultimately result in improvements in prevention, early detection, and treatment.

## Introduction

Breast cancers vary greatly in clinical behavior, morphological appearance, and molecular alterations. Accumulating epidemiologic data also suggest that different types of breast cancers have different risk factor profiles and thus might result from different etiologic pathways (which might be shared by different tumor types or be type specific). Notably, age-specific incidence rates [1] and the strength of the associations with known risk factors for breast cancer [2]–[4] differ by clinically important tumor characteristics. Evidence that genetic factors can also influence tumor type is provided by the fact that carriers of highly penetrant mutations in *BRCA1* are more likely to be diagnosed with basal breast tumors which are estrogen receptor (ER) negative, progesterone receptor (PR) negative and HER2 negative [5]. This raises the possibility that other susceptibility loci may also be associated with specific subtypes of breast cancer.

We recently performed a two-stage genome-wide association study (GWAS) in 4,398 breast cancer cases and 4,316 controls, followed by a third stage in 21,860 cases and 22,578 controls from 22 studies, identifying single nucleotide polymorphisms (SNPs) in 5 loci associated with breast cancer risk [6]. Of the five loci identified, 4 were within genes or linkage disequilibrium (LD) blocks containing genes, including: 1) rs2981582 in the *FGFR2* gene coding for a receptor tyrosine kinase that plays an important role in mammary gland development [7], has been implicated in carcinogenesis [8], and is amplified [9]–[11] or over-expressed [12] in up to 10% of breast tumors; 2) rs3803662 in a LD block containing *TNRC9* (also known *TOX3*) and the hypothetical gene *LOC643714*; 3) rs889312 in a LD block containing *MAP3K1* and two hypothetical genes (*MGC33648* and mesoderm induction early response 1, family member 3 (*MIER3*)); and 4) rs3817198 in the *LSP1* gene. The fifth SNP (rs13281615) lies on a region of 8q24 that does not contain known genes, but has multiple independent variants associated with prostate [13],[14] and colorectal [15]–[18] cancer risk. Two additional genome wide association studies also recently identified SNPs in *FGFR2* [19] and *TNRC9* [20] as breast cancer susceptibility loci.

We used the large data resource provided by the Breast Cancer Association Consortium (BCAC) to evaluate the hypothesis that tumor characteristics modify the association between breast cancer risk and the low penetrant susceptibility loci recently identified [6]. Determining whether breast cancer risk factors are linked to tumors with specific clinical presentations, pathologic characteristics or mechanisms of development may provide a gateway for developing tailored prevention and early detection strategies. In addition, we evaluated whether these genetic factors affect overall survival after diagnosis of breast cancer, either independently or through their association with tumor characteristics of clinical importance.

## Materials and Methods

### Study Populations

Cases and controls were identified through 21 case-control studies in Europe, North America, South-East Asia and Australia, participating in the BCAC (see Table S1 for description of study populations). All of these studies, except for two Germany studies (Mammary Carcinoma Risk Factor Investogation (MARIE), Genetic Epidemiology Study of Breast Cancer by Age 50 (GESBC)), were included in our previous publication [6] (the ORIGO study was previously referred to as LUMCBCS), and provided information on disease status, age at diagnosis/enrollment, ethnic group (European, Asian, other), first degree family history of breast cancer and bilaterality of breast cancer. Twenty studies with a total of 23,839 invasive breast cancer cases and 26,928 controls also provided data on tumor characteristics (i.e. histopathologic subtype, ER and PR receptor status, tumor size, grade, nodal involvement or stage; see Table S2 for data sources). Of these, 800 cases and 655 controls were excluded from analyses because of failures in genotyping quality control (see details under *Genotyping*) or because they belonged to “other” ethnic groups with few subjects. Data on survival after diagnosis was available for 13,527 cases participating in 13 studies (after excluding failures in genotype QC and “other” ethnicities), including the USRT study, which lacked data on tumor characteristics (Table S4). Overall, 95.6% of cases and 96.7% of controls were of European origin. The mean ages were 56 years for cases and 57 years for controls.

The distribution of tumor characteristics by study among the 23,039 ( = 23839-800) cases from 20 studies with pathology information is shown in Table S4. Data pertaining to the first tumor detected were used for women with bilateral disease. Data related to histological subtype was available for 86% of the cases (18 studies), ER status for 74% (20 studies), PR status for 62% (18 studies), tumor grade of differentiation for 70% (17 studies), nodal involvement for 65% (17 studies), tumor size for 35% (9 studies), and stage at diagnosis for 68% (11 studies). A total of 1,487 of the 23,039 cases were excluded because they had missing information on all tumor characteristics, leaving 21,552 cases and 26,273 controls of European or Asian origin available for analyses by tumor characteristics. The actual number of cases and controls included in each analysis, after excluding missing genotype data, is shown in the tables.

### Genotyping

Genotyping procedures have previously been described [6]. All studies genotyped for the five SNPs with the exception of rs3803662 that was not genotyped in the KConFab study, and rs13281615 that was not genotyped in KConFab and MARIE studies. Any sample that could not be scored on 20 percent of the SNPs attempted was excluded from analysis. We also removed data for any center/SNP combination for which the call rate was less than 90 percent. In any instances where the call rate was 90–95 percent, the clustering of genotype calls was re-evaluated by an independent observer to determine whether the clustering was sufficiently clear for inclusion. We also eliminated all of the data for a given SNP/center where the reproducibility in duplicate samples was <97 percent, or where there was marked deviation from Hardy-Weinberg equilibrium in the controls (p<.00001).

### Statistical Analyses

Polytomous logistic regression was used to estimate adjusted odds ratios (OR) and associated 95 percent confidence intervals (CI) as measures of association between genotypes and risk of breast cancer subtypes (comparing case subtypes to all controls). All models included terms for study (dummy variables). Further adjustment for age at diagnosis/enrollment did not substantially influence OR estimates (data not shown). We estimated the association for each SNP in terms of genotype-specific ORs and per-allele ORs (assuming a log-additive model). Heterogeneity between genotype odds ratios for different tumor subtypes was assessed using logistic regression analyses restricted to cases (case-only analyses) with the tumor characteristic as the outcome variable. For tumor subtypes with more than two levels (i.e. grade, size, stage), we used a polytomous logistic regression model constraining the effect size to increase linearly across levels (e.g. the parameter for grade 3 vs grade1 = 2*grade2 vs grade1). To evaluate which of several correlated tumor features was most important in determining genotype associations, we fitted logistic regression models with one of the tumor features as the outcome and the genotype and other tumor features as explanatory variables.

Survival analyses were based on 13,527 breast cancer cases from 13 studies with available follow-up data. Univariate analyses for each SNP were carried out by estimating Kaplan-Meier survival curves stratified by genotypes, and by fitting Cox proportional hazards regression models adjusting for study and left-truncating at date of blood draw to allow for inclusion of prevalent cases. This provides an unbiased estimate of the hazard ratio provided that the proportional hazards assumption holds. The assumption of proportional hazards was tested by visual inspection of standard log-log plots and analytically using Schoenfeld residuals. Time at risk was calculated from the date of blood sample draw to date of death or last follow-up, whichever date came first. Follow-up for all cases was censored at 10 years after the initial diagnosis because the number of cases with longer time of follow-up was relatively small, and they are likely to be a selected group of patients due to lost to follow up. A total of 1,584 deaths occurred during eligible follow-up. We also carried out analyses adjusting for other determinants of survival (age at diagnosis (continuous), ER and PR status (each dichotomous), grade (ordinal), tumor size (continuous) and nodal involvement (dichotomous)). Survival analyses were conducted for all cases combined, and separately for ER-positive and ER-negative cases. Data were analyzed using STATA v.9. for Windows (College Station, TX).

The main conclusions from our analyses are based on comparisons of five SNPs with seven correlated tumor characteristics (i.e. ER, PR, grade, nodes, size, histology and stage at diagnosis) and survival after diagnosis. We have used a permutation adjustment procedure [21] to correct P values for these 40 hypothesis tests. The tumor characteristics were permuted in a group with respect to the SNPs. In this procedure, the outcomes (i.e. tumor characteristics) were randomly assigned against the SNPs while retaining the correlation structure of the outcomes. We performed 1000 permutations to obtain the empirical distribution of P values under the null hypothesis of no association. Multiple-comparisons-permutation-adjusted P values for each of the 40 tests were calculated as the proportion of P values equal or smaller than the observed P value.

### Entrez Gene Accession Numbers

*GFR2*: 2263

*TNRC9* or *TOX3*: 27324

*MAP3K1*: 4214

*MIER3*: 166968

*LSP1*: 4046

*v-myc myelocytomatosis viral oncogene homolog (avian) (MYC)*: 4609

## Results

### Association between SNPs and Breast Cancer Risk by Tumor Subtypes

**Figure 1:**
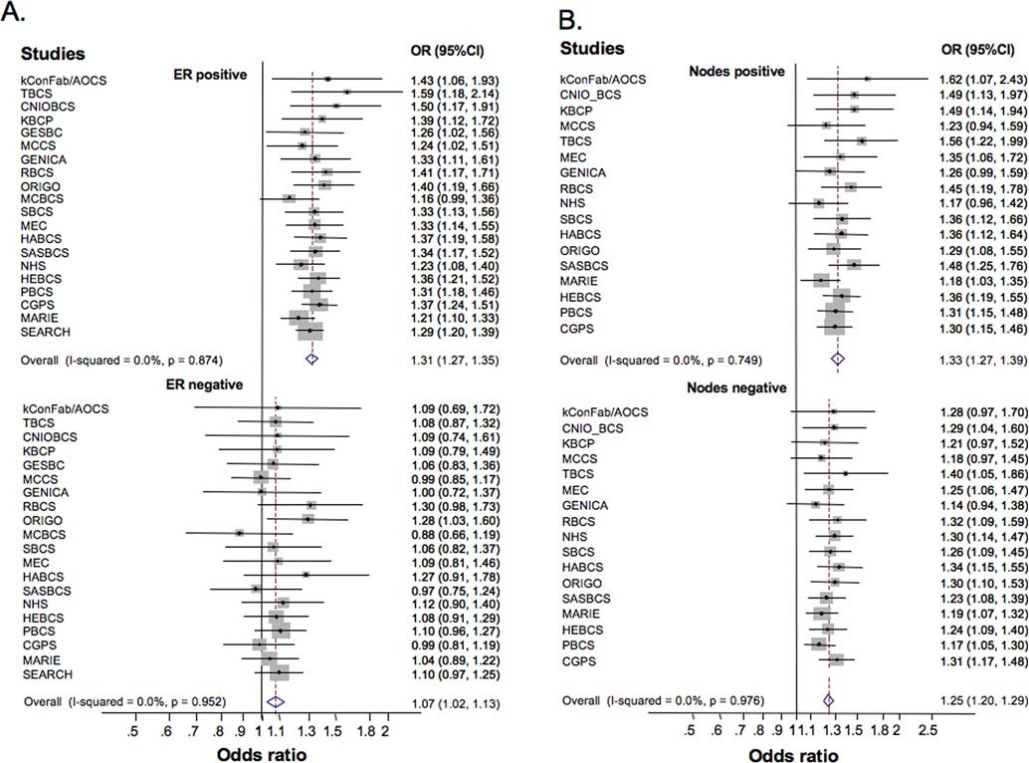
Per-allele odds ratios (ORs) and 95% confidence intervals (CIs) for the association between *FGFR2* (rs2981582) and breast cancer by study. A. stratified by ER status, B. stratified by axillary node involvement. Studies are weighted and ranked according to the inverse of the variance of the log OR estimate for ER-positive (A) or node positive (B) tumors. P for study heterogeneity were 0.84 and 0.96, for the association with ER-positive and negative disease, respectively; and 0.64 and 0.97 for node positive and negative diseases, respectively. See Table S1 for description of the studies and acronyms.

Minor allele frequencies and estimates for the association between the five SNPs evaluated and overall breast cancer risk are shown in Table S5. Stratification of tumors by ER status indicated that rs2981582 in *FGFR2* had a stronger association with ER-positive (per-allele OR (95% CI) = 1.31 (1.27–1.36)) than ER-negative tumors (1.08 (1.03–1.14); P for heterogeneity of ORs = 10^−13^; Table 1; Figure 1 panel A; see Table S6 for estimates by ethnicity). Women with the homozygous variant genotype (present in 14% of controls) had a risk of ER-positive tumors 1.74 (95%CI = 1.63–1.85) times higher than those with the common homozygous genotype (present in 39% of controls) (Table 1). The difference in ORs between ER-positive and ER-negative tumors is consistent across studies (Figure 1 panel A), and it is highly significant even after permutation adjustment for multiple comparisons (P<0.001). The rs2981582 association was also stronger for other tumor characteristics associated with ER status, i.e. PR expression (P = 10^−5^) and lower grade (P = 10^−8^; Table 2; Tables S7, S8). The associations of rs2981582 with ER, PR and grade were significant after permutation adjustment for multiple comparisons (P≤0.001). The modification by ER status remained statistically significant after adjustment for PR status and grade (P = 0.002) based on data from those studies with information on all three tumor characteristics (16 studies including 10,951 cases). On the other hand, the evidence for associations with PR status became non-significant after adjustment for ER status (P = 0.45). The association with grade (Table 2) remained statistically significant after adjustment by ER status (P = 0.003), and after further adjustment for PR status (P = 0.030). Grouping tumors as ER and PR negative versus ER and/or PR positive tumors did not result in further discrimination of risks (data not shown).

**Table 1:** Per-allele odds ratios for breast cancer risk by estrogen receptor status.

Locus			ER-positive cases			ER-negative cases			Observed	Adjusted
SNP	Genotype	Controls	N	OR*	95% CI	N	OR*	95% CI	P**	P***
*FGFR2*	GG	10,056	4,043	1.00		1,378	1.00			
rs2981582	AG	12,255	6,390	1.28	1.22–1.35	1,828	1.08	1.00–1.16		
	AA	3,747	2,636	1.74	1.63–1.85	607	1.18	1.06–1.30		
	*per allele*			1.31	1.27–1.36		1.08	1.03–1.14	10^−13^	<0.001
*TNRC9*	GG	13,295	5,970	1.00		1,789	1.00			
rs3803662	AG	9,705	5,553	1.25	1.19–1.31	1,579	1.16	1.08–1.25		
	AA	2,026	1,451	1.48	1.37–1.60	397	1.28	1.13–1.45		
	*per allele*			1.23	1.19–1.27		1.14	1.09–1.21	0.015	0.43
*MAP3K1*	TT	13,447	6,352	1.00		1,912	1.00			
rs889312	GT	10,480	5,474	1.12	1.07–1.17	1,539	1.03	0.96–1.11		
	GG	2,154	1,271	1.26	1.17–1.36	370	1.20	1.06–1.36		
	*per allele*			1.12	1.09–1.16		1.07	1.01–1.13	0.11	0.99
8q24	AA	7,650	3,721	1.00		1,158	1.00			
rs13281615	AG	10,682	5,681	1.11	1.06–1.17	1,603	0.99	0.91–1.07		
	GG	3,773	2,298	1.29	1.21–1.38	623	1.09	0.98–1.21		
	*per allele*			1.13	1.10–1.17		1.03	0.98–1.09	0.001	0.038
*LSP1*	AA	12,695	6,304	1.00		1,867	1.00			
rs381798	AG	10,995	5,485	1.04	0.99–1.09	1,587	1.01	0.94–1.09		
	GG	2,322	1,281	1.19	1.10–1.28	363	1.13	1.00–1.27		
	*per allele*			1.07	1.04–1.11		1.04	0.99–1.10	0.31	1.00

*: ORs adjusted for study.

**: P for heterogeneity calculated from case-only analyses adjusted for study.

***: Permutation adjusted P for heterogeneity

**Table 2:** Odds ratios for breast cancer risk by tumor grade*.

Locus			Grade 1			Grade 2			Grade 3			Obs.	Adj.
SNP	Genotype	Controls	N	OR**	95% CI	N	OR**	95% CI	N	OR**	95% CI	P***	P****
*FGFR2*	GG	8916	979	1.00		2368	1.00		1686	1.00			
rs2981582	AG	10988	1627	1.32	1.21–1.43	3879	1.31	1.24–1.39	2344	1.12	1.04–1.20		
	AA	3389	696	1.83	1.64–2.03	1521	1.68	1.55–1.81	853	1.32	1.20–1.44		
	*per allele*			1.35	1.28–1.42		1.30	1.25–1.35		1.14	1.09–1.19	10^−8^	<0.001
*TNRC9*	GG	12073	1497	1.00		3620	1.00		2382	1.00			
rs3803662	AG	8570	1436	1.31	1.21–1.42	3285	1.26	1.19–1.33	1978	1.15	1.08–1.23		
	AA	1648	340	1.49	1.31–1.70	775	1.45	1.32–1.59	471	1.33	1.19–1.49		
	*per allele*			1.25	1.19–1.33		1.22	1.18–1.27		1.16	1.10–1.21	0.018	0.50
*MAP3K1*	TT	12218	1650	1.00		3890	1.00		2478	1.00			
rs889312	GT	9316	1371	1.12	1.04–1.21	3217	1.11	1.05–1.17	1997	1.07	1.00–1.15		
	GG	1767	267	1.18	1.03–1.36	679	1.26	1.15–1.39	429	1.23	1.09–1.38		
	*per allele*			1.10	1.04–1.17		1.12	1.07–1.16		1.09	1.04–1.15	0.91	1.00
8q24	AA	6794	940	1.00		2163	1.00		1505	1.00			
rs13281615	AG	9335	1456	1.15	1.05–1.26	3266	1.11	1.04–1.18	2063	1.01	0.94–1.09		
	GG	3185	593	1.42	1.27–1.59	1329	1.35	1.25–1.47	783	1.14	1.03–1.26		
	*per allele*			1.18	1.12–1.25		1.16	1.11–1.20		1.06	1.01–1.11	10^−4^	0.016
*LSP1*	AA	11239	1549	1.00		3669	1.00		2287	1.00			
rs381798	AG	9923	1409	1.07	0.99–1.16	3314	1.04	0.99–1.10	2145	1.09	1.02–1.16		
	GG	2100	333	1.24	1.09–1.41	792	1.21	1.10–1.32	476	1.17	1.04–1.30		
	*per allele*			1.10	1.04–1.16		1.08	1.04–1.12		1.08	1.03–1.14	0.77	1.00

*: Analyses excluded data from three studies (MEC, NHS and TBCS) without information on tumor grade.

**: Per-allele OR adjusted for study.

***: P value for heterogeneity of ORs from case-only analyses adjusted by study.

****: Permutation adjusted P for heterogeneity

The association of rs2981582 with breast cancer risk tended to be stronger for patients with positive (per-allele OR (95% CI) = 1.33 (1.27–1.39)) compared to negative (1.25 (1.20–1.29)) nodal involvement (P = 0.013; Table 3; see Table S9 for estimates by ethnicity). Although differences were small and not significant after permutation adjustment for multiple comparisons (P = 0.41), they were consistent across studies (Figure 1, panel B). Nodal involvement was correlated with tumor grade and size, and the association between nodal involvement and rs2981582 among cases remained significant (P = 0.010) after adjustment for these tumor characteristics in 9 studies with 6,204 cases. Nodal involvement and ER status were independently associated with rs2981582 in 12,374 cases from 17 studies with data on these two factors (P value for node association with rs2981582 adjusted by ER = 0.022; P = 0.75 after adjusting for multiple testing). rs2981582 showed the strongest association with node positive ER-positive tumors (29% of all tumors; per-allele OR (95% CI) = 1.37 (1.29–1.44)), followed by node negative ER-positive tumors (48% of all tumors; 1.30 (1.25–1.36)) and node positive ER-negative tumors (10% of all tumors; 1.18 (1.09–1.29) (Table S10). No increase in risk was observed for node negative ER-negative tumors (13% of tumors; 1.05 (0.97–1.13).

**Table 3:** Odds ratios for breast cancer risk by lymph node involvement*.

Locus			Cases with negative nodes			Cases with positive nodes			Observed	Adjusted
SNP	Genotype	Controls	N	OR**	95% CI	N	OR**	95% CI	P***	P****
*FGFR2*	GG	7494	3031	1.00		1683	1.00			
rs2981582	AG	9021	4539	1.23	1.16–1.30	2753	1.32	1.23–1.42		
	AA	2746	1734	1.56	1.44–1.68	1104	1.77	1.61–1.94		
	*per allele*			1.25	1.20–1.29		1.33	1.27–1.39	0.013	0.41
*TNRC9*	GG	9762	4234	1.00		2470	1.00			
rs3803662	AG	7180	3930	1.22	1.16–1.29	2391	1.26	1.18–1.34		
	AA	1551	1028	1.36	1.24–1.49	639	1.35	1.21–1.51		
	*per allele*			1.19	1.14–1.24		1.20	1.14–1.26	0.78	1.00
*MAP3K1*	TT	9867	4547	1.00		2707	1.00			
rs889312	GT	7753	3864	1.11	1.05–1.17	2291	1.11	1.04–1.19		
	GG	1656	919	1.23	1.12–1.35	572	1.32	1.18–1.48		
	*per allele*			1.11	1.06–1.15		1.14	1.08–1.19	0.31	1.00
8q24	AA	5257	2607	1.00		1634	1.00			
rs13281615	AG	7316	3921	1.11	1.04–1.18	2372	1.08	1.00–1.17		
	GG	2741	1635	1.23	1.13–1.33	961	1.22	1.11–1.35		
	*per allele*			1.11	1.07–1.15		1.10	1.05–1.16	0.95	1.00
*LSP1*	AA	9357	4531	1.00		2708	1.00			
rs381798	AG	8148	3917	1.03	0.98–1.09	2318	1.05	0.98–1.12		
	GG	1749	866	1.11	1.02–1.22	534	1.20	1.07–1.34		
	*per allele*			1.05	1.01–1.09		1.08	1.03–1.13	0.38	1.00

*: Analyses excluded data from three studies (GESBC, MCBCS and SEARCH) without information on node involvement.

**: Per-allele OR adjusted for study.

***: P value for heterogeneity of ORs from case-only analyses adjusted by study.

****: Permutation adjusted P for heterogeneity.

The association of rs13281615 in 8q24 with risk was also stronger for ER-positive compared to ER-negative tumors (P = 0.001; Table 1; Figure S1). This SNP also showed a stronger association with PR-positive than negative tumors (P = 0.011; Table S7) and lower tumor grade (P = 10^−4^; Table S8). Only the associations of rs13281615 with ER and grade, but not with PR, were significant after permutation adjustment for multiple comparisons (P = 0.037, 0.016, 0.35, respectively). The associations with ER and grade were significant after adjustment for each other (P = 0.029 for ER adjusted for grade and 0.035 for grade adjusted for ER in 15 studies with 11,419 cases with data on ER and grade), while the association with PR was not significant after ER adjustment (P = 0.31). The association of rs3803662 in *TNRC9* and breast cancer was also significantly modified by ER status (P = 0.015; Table 1)) and grade (P = 0.018; Table 2). However, these differences were not significant after permutation adjustment for multiple comparisons (P = 0.42 for ER, 0.50 for grade), or when adjusted for each other in 16 studies with 13,075 cases with data on ER and grade (P = 0.11 for ER adjusted by grade, and P = 0.37 for grade adjusted by ER).

Three SNPs (rs2981582 in *FGFR2*, rs3803662 in *TNRC9* and rs889312 in *MAP3K1*) were associated with significant increases in risk of ER-negative tumors (Table 1), although to a lesser extent than ER-positive tumors. Of these SNPs, rs3803662 showed the strongest association with ER-negative tumors: women with the homozygous variant genotype (present in 8% of controls) had a 1.28 (95%CI = 1.13–1.45) higher risk of developing ER-negative disease than women with the common homozygous genotype (present in 53% of controls) (Table 1).

No significant modification of the ORs was observed for stage at initial diagnosis for any of the 5 loci (Table S13). Of note, rs889312 in *MAP3K1* and rs3817198 in *LSP1* were not associated with any of the tumor characteristics (Tables S6, S7, S8, S9 and S11, S12, S13). Modification of ORs by tumor characteristics generally followed similar patterns for Europeans and Asians, although the number of Asians was substantially smaller, and thus most differences by tumor type were not statistically significant. An exception was the presence of stronger associations with larger tumors for rs889312 in *MAP3K1* (P = 0.015; Table S11) in Asian but not in European populations.

### Survival Analyses

The average time at risk (i.e. date of blood sample draw to date of death, last follow-up or censored time, whichever date came first) among 13,527 breast cancer patients in 13 studies was 6.0 years with a range between <1 and 10 years in individual studies. Cases were followed-up for a total of 54,716 person-years with the occurrence of 1,515 deaths from any cause (Table S3). As expected, survival was poorer for patients with ER negative, PR negative, higher grade and larger tumors and in patients with positive nodes (Figure S2). No differences in survival by genotype were found, except for possibly better survival in patients with the variant allele in rs13281615 at 8q24 (unadjusted per-allele HR (95%CI) = 0.90 (0.83–0.97), P = 0.009; Table 4). This association was no longer significant after adjustment for ER status, grade and age at diagnosis (adjusted HR = 0.92 (0.83–1.01), Table 4). Weaker evidence of poorer survival was observed in patients diagnosed with ER-negative tumors carrying the variant allele in rs3803662 (P = 0.071). This association was independent of grade and age at diagnosis (adjusted per-allele HR (95%CI) = 1.19 (0.98–1.44); Table 4; Figure S3).

**Table 4:** Multivariate Cox proportional hazards analysis of genetic polymorphisms in relation to overall survival following breast cancer diagnosis, by ER status*.

		Unadjusted**				Adjusted****		
Locus	SNP	HR**	95% CI	Obs P	Adj P***	HR**	95% CI	Obs P
All tumors								
*FGFR2*	rs2981582	0.98	0.91–1.05	0.56	1.00	1.01	0.92–1.11	0.82
*TNRC9*	rs3803662	1.05	0.96–1.15	0.26	1.00	1.06	0.95–1.19	0.31
*MAP3K1*	rs889312	1.02	0.95–1.11	0.54	1.00	1.03	0.93–1.15	0.52
8q24	rs13281615	0.90	0.83–0.97	0.009	0.32	0.92	0.83–1.01	0.084
*LSP1*	rs381798	0.99	0.92–1.07	0.88	1.00	1.03	0.93–1.14	0.55
ER positive tumors								
*FGFR2*	rs2981582	1.00	0.90–1.11	0.98		1.03	0.92–1.16	0.62
*TNRC9*	rs3803662	1.02	0.89–1.16	0.82		1.00	0.86–1.15	0.97
*MAP3K1*	rs889312	0.99	0.88–1.11	0.82		0.99	0.87–1.13	0.91
8q24	rs13281615	0.88	0.78–0.99	0.039		0.89	0.78–1.01	0.068
*LSP1*	rs381798	1.09	0.98–1.23	0.12		1.07	0.94–1.21	0.32
ER negative tumors								
*FGFR2*	rs2981582	0.99	0.85–1.15	0.87		0.98	0.84–1.14	0.78
*TNRC9*	rs3803662	1.19	0.99–1.43	0.071		1.19	0.98–1.44	0.076
*MAP3K1*	rs889312	1.15	0.98–1.35	0.08		1.11	0.94–1.32	0.22
8q24	rs13281615	0.95	0.81–1.11	0.48		0.96	0.82–1.13	0.64
*LSP1*	rs381798	0.95	0.81–1.11	0.49		0.97	0.82–1.15	0.74

*: Analyses by ER status included data from 12 studies with information on vital status and ER status (CGPS, CNIO-BCS, HABCS, HEBCS, KBCP, kConFab, LUMCBCS, MCCS, PBCS, SASBCS, SBCS, SEARCH).

**: Per-allele hazard ratios (HR) and observed P values are adjusted for study. Allele changes are (common>rare based on frequencies in European populations): G>A for rs2981582; G>A for rs3803662; T>G for rs889312; A>G for rs13281615 and A>G for rs381798.

***: Permutation adjusted P values.

****: Per-allele hazard ratios (HR) and observed P values are adjusted for study, age at diagnosis (continuous), ER status and grade (continuous). Analyses limited to 11 studies with ER and grade information (CGPS, CNIO-BCS, HABCS, HEBCS, KBCP, LUMCBCS, MCCS, PBCS, SASBCS, SBCS, SEARCH).

The P values for the interaction between ER status and genotype adjusted for study, grade and age at diagnosis are: 0.60, 0.15, 0.29, 0.45, 0.38 for rs2981582, rs3803662, rs889312, rs13281615, rs381798, respectively.

## Discussion

This report has demonstrated that common genetic variants that predispose to breast cancer may also be linked to clinically important characteristics of tumors, including size, grade, ER and PR status, and nodal involvement. A major strength of our study is the large sample size after pooling data from multiple studies with information on tumor characteristics, which allowed for precise estimates of relative risk by most tumor subtypes.

The most notable finding was for rs2981582 located in *FGFR2*, which showed a stronger association with ER-positive than ER-negative tumors (P = 10^−13^), with lower than higher grade tumors (P = 10^−8^) and with node positive than negative tumors (P = 0.013). This SNP was significantly associated only with ER-negative tumors that involved lymph nodes. rs2981582 also showed stronger associations with PR-positive tumors but this association was not independent of ER status. The stronger association with ER-positive tumors is supported by previous observations indicating that *FGFR2* is involved in estrogen-related breast carcinogenesis [22]–[25], and that levels of expression of the receptor are higher in ER-positive than ER-negative cell lines [26] and tumors [27].

We have shown previously that the causative variant in *FGFR2* is likely to be one of six variants correlated with rs2981582 in a region of intron 2 containing multiple transcription factor binding sites. This suggests that the association with breast cancer risk may be mediated through differential levels of *FGFR2* expression [6]. In addition, as *FGFR2* has been shown to be overexpressed or amplified only in a small percentage of breast cancers [9],[10],[24], it is possible that the association with breast cancer risk could be stronger and more clinically relevant for the small subset of tumors that express high levels of the receptor. Epidemiological studies stratifying by levels of tumor expression of *FGFR2* , its ligands or co-factors may clarify the role of *FGFR2* variation in breast cancer risk.

rs13281615 in 8q24 was also more strongly associated with ER-positive and lower grade tumors, although differences were smaller than for rs2981582 in *FGFR2*. Other independent variants in the 8q24 region which does not contain known genes, have been associated with prostate cancer risk [11],[13],[14]; however, the mechanisms for the associations with these cancers are unknown. A recent GWAS comprising five studies with 4,533 cases and 17,513 controls (including samples from the MEC study in this report) showed the risk from rs3803662 in *TNRC9* to be significantly greater in ER-positive tumors [20]. Our data also showed a stronger association with ER-positive than ER-negative tumors, but the difference was smaller and not statistically significant based on the analysis of 12,832 cases and 22,356 controls from 18 studies. Moreover, this SNP showed the strongest association with ER-negative disease among the five evaluated. Future studies might reveal stronger associations between these SNPs and tumor subtypes defined by different markers, or perhaps molecular subtypes previously defined by gene expression profiling [28],[29].

It is possible that our study preferentially detected SNPs associated with ER-positive rather than ER-negative disease, since the majority of breast cancer cases in the initial GWAS were ER positive. This raises the possibility that genome-wide association studies focusing on the less common breast tumor subtypes may identify different risk loci. Of particular importance might be SNPs identified in studies of basal tumor subtypes since they are often clinically aggressive and difficult to treat effectively, and have been associated with germline mutations in *BRCA1* [5],[28].

Differences in the design, source of information on tumor characteristics and criteria to classify tumors across studies could lead to heterogeneity of findings by study, which limits the ability to detect modification of genotype associations by tumor characteristics. However, findings were generally consistent across studies (Figure 1 and Figure S1), particularly for the *FGFR2* (rs2981582) association by ER status, arguing for the robustness of our results. Genotype associations with risk of breast cancer were similar for subjects with and without information on tumor characteristics (data not shown), indicating that missing information is unlikely to substantially affect our results.

None of the five SNPs included in this report had a significant association with overall survival independent of their associations with known prognostic factors. Only rs13281615 in 8q24 was significantly associated with survival in unadjusted analyses. Adjustment for ER status and grade resulted in a weaker, non-significant association with survival, suggesting that the increased survival is partially mediated through the higher probability of developing tumors with favorable prognostic characteristics. Any SNP effect on overall survival, if mediated through known prognostic tumor characteristics, would be expected to be small because of the small magnitude of risk differences by tumor subtypes; thus the power to detect a difference in survival would be low. For instance, at a type I error rate of 0.01, the power to detect alleles with minor allele frequency (MAF) = 0.3 that confer a per-allele HR of 1.1 is only 40%. Another limitation of the survival analyses is that relapse or disease-specific mortality data were not available for most studies and use of all cause mortality as the end point may further reduce power. Finally, any impact of SNPs on survival may interact with treatment, particularly adjuvant chemotherapy, or other determinants of survival such obesity. However, this could not be evaluated since information on treatment or other factors affecting survival was not available.

We have shown that there is heterogeneity in the risk of different tumor types for common breast cancer susceptibility alleles, with the clearest difference being in the relative risk of ER-positive and ER-negative tumors for the variant in *FGRF2*. Other differences were observed, however, the weight of evidence was weaker and needs further confirmation in additional studies. These findings provide further support for the notion that ER-negative and ER-positive tumors result from different etiologic pathways, rather than different stages of tumor evolution within a common carcinogenic pathway [30]. The magnitude of the observed differences is small, and by themselves these findings are unlikely to have any immediate clinical implications. However, the observed differences provide clues to the biological mechanisms that underpin tumor heterogeneity, which may ultimately lead to improved treatment and prevention.

## Supporting Information

Figure S1 Per-allele odds ratios (ORs) and 95% confidence intervals (CIs) for the association between SNPs and breast cancer by study, stratified by ER status. Studies are weighted and ranked according to the inverse of the variance of the log OR estimate for ER-positive tumors. P for study heterogeneity for the association with ER-positive/ER-negative disease, respectively, were 0.77/0.99 for rs3803662; 0.72/0.29 rs889312; 0.55/0.31 for rs13281615; and 0.55/0.46 for rs3817198. See Table S1 for description of the studies and acronyms.
(0.30 MB DOC)

Figure S2 Kaplan-Meier plot showing survival after stratifying for estrogen and progesterone receptor status, histological grade, tumor size, nodal status, and histopathology.
(0.15 MB DOC)

Figure S3 Kaplan-Meier plots showing survival in different genotypes of (A.) rs3803662 inTNRC9 and (B.) rs13281615 in 8q24 among cases diagnosed with ER-positive and ER-negative tumors.
(0.14 MB DOC)

Table S1 Summary of the 21 breast cancer case studies used in the analyses for tumor characteristics and survival.
(0.13 MB DOC)

Table S2 Information content, sources of information for tumor characteristics and survival data, and relevant publications for the 21 participating studies.
(0.08 MB DOC)

Table S3 Number of cases, person-years at risk, number of deaths, mortality rate (MR), and 95 percent confidence intervals (95%CI) in the 13 studies with follow-up information.
(0.05 MB DOC)

Table S4 Distribution of tumor characteristics among 23,039 invasive breast cancer cases in the 20 participating studies with information on tumor.
(0.03 MB DOC)

Table S5 Per-allele odds ratios for the association between SNPs and invasive breast cancer risk in 20 studies included in the assessment of tumor characteristics in this report.
(0.05 MB DOC)

Table S6 Per-allele odds ratios for breast cancer risk by estrogen receptor status, stratified by ethnicity.
(0.07 MB DOC)

Table S7 Per-allele odds ratios for breast cancer risk by progesterone receptor status, stratified by ethnicity.
(0.07 MB DOC)

Table S8 Per-allele odds ratios for breast cancer risk by grade, stratified by ethnicity.
(0.07 MB DOC)

Table S9 Per-allele odds ratios for breast cancer risk by nodal status, stratified by ethnicity.
(0.07 MB DOC)

Table S10 Per-allele odds ratios for the association between *FGFR2* rs2981582 and breast cancer risk by ER and nodal status.
(0.03 MB DOC)

Table S11 Per-allele odds ratios for breast cancer risk by tumor size, stratified by ethnicity.
(0.09 MB DOC)

Table S12 Per-allele odds ratios for breast cancer risk by histopathogic subtypes, stratified by ethnicity.
(0.06 MB DOC)

Table S13 Per-allele odds ratios for breast cancer risk by stage at diagnosis, stratified by ethnicity.
(0.08 MB DOC)
