# Supplementary material for: Heterogeneity of Breast Cancer Associations with Five Susceptibility Loci by Clinical and Pathological Characteristics
Source: PLoS Genet. 2008 Apr 25;4(4):e1000054. doi: 10.1371/journal.pgen.1000054 (PMC2291027; doi:10.1371/journal.pgen.1000054)
Supplement: Figure S1 — Per-allele odds ratios (ORs) and 95% confidence intervals (CIs) for the association between SNPs and breast cancer by study, stratified by ER status. Studies are weighted and ranked according to the inverse of the variance of the log OR estimate for ER-positive tumors. P for study heterogeneity for the association with ER-positive/ER-negative disease, respectively, were 0.77/0.99 for rs3803662; 0.72/0.29 rs889312; 0.55/0.31 for rs13281615; and 0.55/0.46 for rs3817198. See Table S1 for description of the studies and acronyms. (0.30 MB DOC) [file pgen.1000054.s001.doc]

Figure S1
