# Supplementary material for: Heterogeneity of Breast Cancer Associations with Five Susceptibility Loci by Clinical and Pathological Characteristics
Source: PLoS Genet. 2008 Apr 25;4(4):e1000054. doi: 10.1371/journal.pgen.1000054 (PMC2291027; doi:10.1371/journal.pgen.1000054)
Supplement: Figure S2 — Kaplan-Meier plot showing survival after stratifying for estrogen and progesterone receptor status, histological grade, tumor size, nodal status, and histopathology. (0.15 MB DOC) [file pgen.1000054.s002.doc]

Figure S2
