# Supplementary material for: Heterogeneity of Breast Cancer Associations with Five Susceptibility Loci by Clinical and Pathological Characteristics
Source: PLoS Genet. 2008 Apr 25;4(4):e1000054. doi: 10.1371/journal.pgen.1000054 (PMC2291027; doi:10.1371/journal.pgen.1000054)
Supplement: Figure S3 — Kaplan-Meier plots showing survival in different genotypes of (A.) rs3803662 inTNRC9 and (B.) rs13281615 in 8q24 among cases diagnosed with ER-positive and ER-negative tumors. (0.14 MB DOC) [file pgen.1000054.s003.doc]

Figure S3
