# Supplementary material for: Heterogeneity of Breast Cancer Associations with Five Susceptibility Loci by Clinical and Pathological Characteristics
Source: PLoS Genet. 2008 Apr 25;4(4):e1000054. doi: 10.1371/journal.pgen.1000054 (PMC2291027; doi:10.1371/journal.pgen.1000054)
Supplement: Table S1 — Summary of the 21 breast cancer case studies used in the analyses for tumor characteristics and survival. (0.13 MB DOC) [file pgen.1000054.s004.doc]

Table S1. Summary of the 21 breast cancer case studies used in the analyses for tumor characteristics and survival

| Study | Abbreviation | Reference | General Setting | Controls | Invasive cases | Age (cases) range | | | Ethnicity* | Genotyping platform(s) |
| --- | --- | --- | --- | --- | --- | --- | --- | --- | --- | --- |
| Copenhagen Breast Cancer Study and Copenhagen General Population Study | CGPS | [1,2] | Denmark (Copenhagen); population-based | 2994 | 1674 | 20 | - | 93 | E | Taqman |
| Spanish National Cancer Centre Breast Cancer Study | CNIO-BCS | [3] | Spain; hospital-based case-control study | 827 | 707 | 23 | - | 86 | E | Taqman |
| Gene Environment Interaction and Breast Cancer in Germany | GENICA | [4,5] | Germany; population-based case-control study | 619 | 597 | 23 | - | 80 | E | Sequenom iPLEX |
| Genetic Epidemiology Study of Breast Cancer by Age 50 | GESBC | [6] | Germany; population-based case-control study | 558 | 555 | 24 | - | 50 | E | TaqMan |
| Hannover Breast Cancer Study | HABCS | [7] | Germany; hospital-based | 1004 | 1063 | 27 | - | 91 | E (99.9%) | Taqman |
| Helsinki Breast Cancer Study | HEBCS | [8,9,10] | Finland; hospital-based case-control study + additional familial cases. Healthy population controls. | 1035 | 1951 | 18 | - | 96 | E | Taqman |
| Kuopio Breast Cancer Project | KBCP | [11] | Finland; hospital-based prospective clinical cohort | 353 | 467 | 17 | - | 92 | E | Taqman |
| Kathleen Cuningham Foundation Consortium for Familial Breast Cancer | KConFab | [12] | Australia and New Zealand; clinic-based recruitment of familial breast cancer patients (cases) | 731 | 297 | 20 | - | 81 | E | Sequenom iPLEX |
|  |  |  | Australia; population-based case-control study of ovarian cancer and population-based cancer-family study of breast cancer (controls only) |  |  |  |  |  |  |  |
| Mammary Carcinoma Risk Factor Investigation | MARIE | [13] | Germany; population-based case-control study | 3194 | 1611 | 50 |  | 75 | E | Sequenom iPLEX |
| Mayo Clinic Breast Cancer Study | MCBCS | [14] | US; clinic-based case-control study | 833 | 783 | 20 | - | 90 | E (99.5%) | Taqman |
| Melbourne Collaborative Cohort Study | MCCS | [15] | Australia: population-based prospective cohort study | 577 | 512 | 41 | - | 83 | E | Taqman |
| Multiethnic cohort | MEC | [16] | US;case-control study nested in prospective cohort | 805 | 846 | 46 | - | 83 | E (52%)  A (48%) | Taqman |
| Nurses Health Study | NHS | [17] | US; case-control nested in prospective cohort | 1636 | 987 | 44 | - | 79 | E | Taqman |
| Leiden University Medical Centre Breast Cancer Study | ORIGO | [18] | Netherlands (Leiden and Rotterdam), case-control hospital-based | 597 | 1180 | 21 |  | 87 | E | Taqman |
| Polish Breast Cancer Study | PBCS | [19] | Poland, Warsaw and Lodz; population-based case-control study | 2331 | 1944 | 24 | - | 75 | E | Taqman |
| Rotterdam Breast Cancer Study | RBCS | [20] | Netherlands (Rotterdam); hospital-based case-control study | 792 | 774 | 22 | - | 84 | E | TaqMan |
| Singapore and Sweden Breast Cancer Study | SASBCS | [21] | Sweden; population-based case-control study | 1459 | 1285 | 49 | - | 75 | E | Sequenom iPLEX, Taqman |
| Sheffield Breast Cancer Study | SBCS | [22,23] | England; hospital-based case-control study | 1132 | 1076 | 28 | - | 92 | E | Taqman |
| SEARCH | SEARCH | [24] | England; population-based case-control study | 4429 | 4263 | 23 | - | 81 | E | Taqman |
| IARC – Thai Breast Cancer Study | TBCS |  | Thailand, hospital based case-control study | 370 | 467 | 17 |  | 81 | A | Taqman |
| U.S. Radiologic Technologist Study | USRT | [25,26] | US (national); nested case–control study within a cohort of approximately 140,000 radiologic technologists | 1018 | 684 | 22 |  | 91 | E (98.1%) | Taqman |

*E: European origin; A: Asian origin
