# Supplementary material for: Heterogeneity of Breast Cancer Associations with Five Susceptibility Loci by Clinical and Pathological Characteristics
Source: PLoS Genet. 2008 Apr 25;4(4):e1000054. doi: 10.1371/journal.pgen.1000054 (PMC2291027; doi:10.1371/journal.pgen.1000054)
Supplement: Table S2 — Information content, sources of information for tumor characteristics and survival data, and relevant publications for the 21 participating studies. (0.08 MB DOC) [file pgen.1000054.s005.doc]

Table S2. Information content, sources of information for tumor characteristics and survival data, and relevant publications for

the 21 participating studies

| **Study*** | **Information source for tumor characteristics** | **Information source for survival data** | **Reference** |
| --- | --- | --- | --- |
| CGPS | Danish Breast Cancer Group | National Danish Cancer Registry National Danish Civil Registration System | [1,2] |
| CNIO-BCS | Data provided by the treating physician | Not available |  |
| GENICA | Review of medical records and histopathology reports | Not available | [27] |
| GESBC | Review of medical records and pathology reports | Not available |  |
| HABCS | Review of histopathology reports from different sources, and review of medical records at the Dept. of Radiation Oncology, Hannover Medical School | Active follow-up at the Department of Radiation Oncology, Hannover Medical School | [28] |
| HEBCS | Review of medical records and histopathology reports | Active follow up of the medical records until 5 years and annual linkage to the nation-wide Finnish Cancer Registry | [10] |
| KConFab | Review of clinical pathology reports | Passive follow up through annual contact with families, matching to the death index, and requests for updated information through biennial newsletters to all participants | [12] |
| KBCP | Review of medical records and histopathology reports (all tumor slides were re-evaluated) | Active follow up of the medical records (last update 12/2006) and linkage to the Finnish Cancer Registry | [11] |
| MARIE | Review of medical records and pathology reports | Not available | [13] |
| MCBCS | Review of medical records and pathology reports | Active follow up of medical records | [14] |
| MCCS | Population cancer registry and review of histopathology reports | Population cancer registry, which receives all death registrations for state of Victoria on a monthly basis and performs annual links to NDI. Linkage to Electoral Register tracks subjects who move inter-state | [15] |
| MEC | SEER tumor registries in Los Angeles and Hawaii | Not available |  |
| NHS | Review of medical records | USA National Death Index |  |
| ORIGO | Review of clinical pathology reports | Active follow-up of medical records | [18] |
| PBCS | Review of medical records, and a surgical pathology form that was completed after clinical sign-out of cases | Active follow up through review of medical records every five years |  |
| RBSC | Review of medical records | Not available |  |
| SASBCS | Review of medical records and histopathology reports | Active follow up of the medical records (last update 2001) and linkage to the nation-wide Swedish Cause of Death Registry | [29] |
| SEARCH | Review of pathology reports and medical records by the cancer registry. | Combination of passive follow-up through national death registrations and active follow up every five years by the cancer registry | [30] |
| TBCS | Review of medical records and histopathology reports | Not available | [31] |
| SBCS | Review of medical records and histopathology reports | Active follow-up by review of medical records and death registration data every five years | [6] |
| USRT | Not available | Survival data are obtained by follow-up through the National Death Index (NDI+) | [25] |

*See definitions of study abbreviations in Table S1
