# Supplementary material for: Heterogeneity of Breast Cancer Associations with Five Susceptibility Loci by Clinical and Pathological Characteristics
Source: PLoS Genet. 2008 Apr 25;4(4):e1000054. doi: 10.1371/journal.pgen.1000054 (PMC2291027; doi:10.1371/journal.pgen.1000054)
Supplement: Table S3 — Number of cases, person-years at risk, number of deaths, mortality rate (MR), and 95 percent confidence intervals (95%CI) in the 13 studies with follow-up information. (0.05 MB DOC) [file pgen.1000054.s006.doc]

Table S3. Number of cases, person-years at risk, number of deaths, mortality rate (MR), and 95 percent confidence intervals (95 %CI) in the 13 studies with follow-up information

| Study | Number of cases | Person-years | Deaths | MR | 95% CI | | |
| --- | --- | --- | --- | --- | --- | --- | --- |
| CGPS | 1,642 | 3,742 | 146 | 0.039 | 0.033 | - | 0.046 |
| CNIO-BCS | 211 | 573 | 6 | 0.010 | 0.005 | - | 0.023 |
| HABCS | 151 | 939 | 22 | 0.023 | 0.015 | - | 0.036 |
| HEBCS | 1,950 | 8,714 | 219 | 0.025 | 0.022 | - | 0.029 |
| KBCP | 461 | 2,518 | 72 | 0.029 | 0.023 | - | 0.036 |
| kConFab | 308 | 755 | 23 | 0.030 | 0.020 | - | 0.046 |
| ORIGO | 489 | 2,085 | 80 | 0.038 | 0.031 | - | 0.048 |
| MCCS | 512 | 3,205 | 76 | 0.024 | 0.019 | - | 0.030 |
| PBCS | 785 | 3,065 | 67 | 0.022 | 0.017 | - | 0.028 |
| SASBCS | 1,285 | 5,286 | 146 | 0.028 | 0.023 | - | 0.032 |
| SBCS | 787 | 1,831 | 89 | 0.049 | 0.039 | - | 0.060 |
| SEARCH | 4,262 | 21,350 | 561 | 0.026 | 0.024 | - | 0.029 |
| USRTS | 684 | 652 | 8 | 0.012 | 0.006 | - | 0.025 |
| Total | 13,527 | 54,716 | 1,515 | 0.028 | 0.026 | - | 0.029 |

*See definitions of study abbreviations in Table S1
