# Supplementary material for: Heterogeneity of Breast Cancer Associations with Five Susceptibility Loci by Clinical and Pathological Characteristics
Source: PLoS Genet. 2008 Apr 25;4(4):e1000054. doi: 10.1371/journal.pgen.1000054 (PMC2291027; doi:10.1371/journal.pgen.1000054)
Supplement: Table S4 — Distribution of tumor characteristics among 23,039 invasive breast cancer cases in the 20 participating studies with information on tumor. (0.03 MB DOC) [file pgen.1000054.s007.doc]

Table S4: Distribution of tumor characteristics among 23,039 invasive breast cancer cases in the 20 participating studies with information on tumor

characteristics*

* A total of 1,487 cases had missing data for all tumor characteristics and thus did not contribute to the analyses. See study description and definitions of abbreviations in Table S1. The USRT study is not shown because tumor information was not available.
