# Supplementary material for: Heterogeneity of Breast Cancer Associations with Five Susceptibility Loci by Clinical and Pathological Characteristics
Source: PLoS Genet. 2008 Apr 25;4(4):e1000054. doi: 10.1371/journal.pgen.1000054 (PMC2291027; doi:10.1371/journal.pgen.1000054)
Supplement: Table S5 — Per-allele odds ratios for the association between SNPs and invasive breast cancer risk in 20 studies included in the assessment of tumor characteristics in this report. (0.05 MB DOC) [file pgen.1000054.s008.doc]

Table S5: Per-allele odds ratios for the association between SNPs and invasive breast cancer risk in 20 studies* included in the assessment of tumor characteristics in this report

| Locus | SNP | MAF** | Cases | Controls | OR*** | 95% CI | | | P |
| --- | --- | --- | --- | --- | --- | --- | --- | --- | --- |
| All populations | |  |  |  |  |  |  |  |  |
| *FGFR2* | rs2981582 | 0.38 | 21,298 | 26,058 | 1.26 | 1.23 | - | 1.30 | <10^-36^ |
| *TNRC9* | rs3803662 | 0.27 | 21,013 | 25,026 | 1.21 | 1.17 | - | 1.24 | 10^-37^ |
| *MAP3K1* | rs889312 | 0.28 | 21,347 | 26,081 | 1.12 | 1.09 | - | 1.15 | 10^-14^ |
| 8q24 | rs13281615 | 0.41 | 19,318 | 22,105 | 1.11 | 1.08 | - | 1.14 | 10^-13^ |
| *LSP1* | rs3817198 | 0.30 | 21,312 | 26,012 | 1.07 | 1.04 | - | 1.10 | 10^-6^ |
| European populations | |  |  |  |  |  |  |  |  |
| *FGFR2* | rs2981582 | 0.38 | 20,410 | 25,314 | 1.26 | 1.23 | - | 1.30 | <10^-36^ |
| *TNRC9* | rs3803662 | 0.27 | 20,126 | 24,299 | 1.21 | 1.17 | - | 1.25 | 10^-36^ |
| *MAP3K1* | rs889312 | 0.28 | 20,455 | 25,331 | 1.12 | 1.09 | - | 1.15 | 10^-13^ |
| 8q24 | rs13281615 | 0.41 | 18,422 | 21,354 | 1.12 | 1.09 | - | 1.15 | 10^-14^ |
| *LSP1* | rs3817198 | 0.31 | 20,426 | 25,266 | 1.07 | 1.04 | - | 1.10 | 10^-6^ |
| Asian populations | |  |  |  |  |  |  |  |  |
| *FGFR2* | rs2981582 | 0.28 | 888 | 744 | 1.32 | 1.14 | - | 1.53 | 10^-4^ |
| *TNRC9* | rs3803662 | 0.55 | 887 | 727 | 1.18 | 1.02 | - | 1.35 | 0.022 |
| *MAP3K1* | rs889312 | 0.55 | 892 | 750 | 1.12 | 0.97 | - | 1.28 | 0.11 |
| 8q24 | rs13281615 | 0.55 | 896 | 751 | 0.98 | 0.86 | - | 1.13 | 0.83 |
| *LSP1* | rs3817198 | 0.14 | 886 | 746 | 1.02 | 0.84 | - | 1.24 | 0.82 |

*Analyses included up to 23,039 cases and 26,273 controls with information on tumor characteristics. Data on rs3803662 in *TNRC9* was missing for the KConFab study, and data on rs13281615 in 8q24 was missing for the KConFab and MARIE studies.

** MAF: minor allele frequency

***Adjusted for study (dummies). Allele changes are (common>rare based on frequencies in European populations): G>A for rs2981582; G>A for rs3803662; T>G for rs889312; A>G for rs13281615 and A>G for rs3817198.
