# Supplementary material for: Heterogeneity of Breast Cancer Associations with Five Susceptibility Loci by Clinical and Pathological Characteristics
Source: PLoS Genet. 2008 Apr 25;4(4):e1000054. doi: 10.1371/journal.pgen.1000054 (PMC2291027; doi:10.1371/journal.pgen.1000054)
Supplement: Table S6 — Per-allele odds ratios for breast cancer risk by estrogen receptor status, stratified by ethnicity. (0.07 MB DOC) [file pgen.1000054.s009.doc]

Table S6. Per-allele odds ratios for breast cancer risk by estrogen receptor status, stratified by ethnicity

|  |  |  | ER-positive cases | | | | |  | ER-negative cases | | | | | Observed | Adjusted |
| --- | --- | --- | --- | --- | --- | --- | --- | --- | --- | --- | --- | --- | --- | --- | --- |
| Locus | SNP | Controls | N | OR* | 95% CI | | |  | N | OR* | 95% CI | | | P** | P*** |
| All populations | |  |  |  |  |  |  |  |  |  |  |  |  |  |  |
| *FGFR2* | rs2981582 | 26,058 | 13,069 | 1.31 | 1.27 | - | 1.36 |  | 3,813 | 1.08 | 1.03 | - | 1.14 | 10^-13^ | <0.001 |
| *TNRC9* | rs3803662 | 25,026 | 12,974 | 1.23 | 1.19 | - | 1.27 |  | 3,765 | 1.14 | 1.09 | - | 1.21 | 0.015 | 0.44 |
| *MAP3K1* | rs889312 | 26,081 | 13,097 | 1.12 | 1.09 | - | 1.16 |  | 3,821 | 1.07 | 1.01 | - | 1.13 | 0.11 | 0.98 |
| 8q24 | rs13281615 | 22,105 | 11,700 | 1.13 | 1.10 | - | 1.17 |  | 3,384 | 1.03 | 0.98 | - | 1.09 | 0.001 | 0.037 |
| *LSP1* | rs3817198 | 26,012 | 13,070 | 1.07 | 1.04 | - | 1.11 |  | 3,817 | 1.04 | 0.99 | - | 1.10 | 0.31 | 1.00 |
| European populations | |  |  |  |  |  |  |  |  |  |  |  |  |  |  |
| *FGFR2* | rs2981582 | 25,314 | 12,608 | 1.31 | 1.27 | - | 1.35 |  | 3,634 | 1.08 | 1.03 | - | 1.14 | 10^-12^ |  |
| *TNRC9* | rs3803662 | 24,299 | 12,513 | 1.23 | 1.19 | - | 1.27 |  | 3,588 | 1.14 | 1.08 | - | 1.20 | 0.012 |  |
| *MAP3K1* | rs889312 | 25,331 | 12,631 | 1.12 | 1.09 | - | 1.16 |  | 3,644 | 1.08 | 1.02 | - | 1.14 | 0.15 |  |
| 8q24 | rs13281615 | 21,354 | 11,233 | 1.14 | 1.10 | - | 1.18 |  | 3,205 | 1.04 | 0.99 | - | 1.10 | 0.002 |  |
| *LSP1* | rs3817198 | 25,266 | 12,612 | 1.07 | 1.04 | - | 1.11 |  | 3,638 | 1.04 | 0.98 | - | 1.10 | 0.28 |  |
| Asian populations | |  |  |  |  |  |  |  |  |  |  |  |  |  |  |
| *FGFR2* | rs2981582 | 744 | 461 | 1.36 | 1.14 | - | 1.63 |  | 179 | 1.18 | 0.92 | - | 1.51 | 0.16 |  |
| *TNRC9* | rs3803662 | 727 | 461 | 1.18 | 1.00 | - | 1.40 |  | 177 | 1.21 | 0.95 | - | 1.54 | 0.92 |  |
| *MAP3K1* | rs889312 | 750 | 466 | 1.10 | 0.93 | - | 1.30 |  | 177 | 0.96 | 0.76 | - | 1.22 | 0.35 |  |
| 8q24 | rs13281615 | 751 | 467 | 1.04 | 0.88 | - | 1.24 |  | 179 | 0.88 | 0.70 | - | 1.11 | 0.32 |  |
| *LSP1* | rs3817198 | 746 | 458 | 0.99 | 0.78 | - | 1.26 |  | 179 | 1.20 | 0.88 | - | 1.65 | 0.60 |  |

*Adjusted for study. Allele changes are (common>rare based on frequencies in European populations): G>A for rs2981582; G>A for rs3803662; T>G for rs889312; A>G for rs13281615 and A>G for rs3817198.

**P value for heterogeneity calculated from case-only analyses adjusting for study.

***Permutation adjusted P value for heterogeneity
