# Supplementary material for: Heterogeneity of Breast Cancer Associations with Five Susceptibility Loci by Clinical and Pathological Characteristics
Source: PLoS Genet. 2008 Apr 25;4(4):e1000054. doi: 10.1371/journal.pgen.1000054 (PMC2291027; doi:10.1371/journal.pgen.1000054)
Supplement: Table S7 — Per-allele odds ratios for breast cancer risk by progesterone receptor status, stratified by ethnicity. (0.07 MB DOC) [file pgen.1000054.s010.doc]

Table S7. Per-allele odds ratios for breast cancer risk by progesterone receptor status, stratified by ethnicity

|  |  |  | PR-positive cases | | | | |  | PR-negative cases | | | | | Observed | Adjusted |
| --- | --- | --- | --- | --- | --- | --- | --- | --- | --- | --- | --- | --- | --- | --- | --- |
| Locus | SNP | Controls | N | OR* | 95% CI | | |  | N | OR* | 95% CI | | | P** | P*** |
| All populations | |  |  |  |  |  |  |  |  |  |  |  |  |  |  |
| *FGFR2* | rs2981582 | 24,587 | 9,428 | 1.29 | 1.25 | - | 1.34 |  | 4,783 | 1.16 | 1.11 | - | 1.21 | 10^-5^ | 0.001 |
| *TNRC9* | rs3803662 | 23,538 | 9,337 | 1.22 | 1.18 | - | 1.27 |  | 4,752 | 1.17 | 1.11 | - | 1.23 | 0.14 | 0.99 |
| *MAP3K1* | rs889312 | 24,590 | 9,435 | 1.13 | 1.09 | - | 1.18 |  | 4,798 | 1.12 | 1.07 | - | 1.18 | 0.67 | 1.00 |
| 8q24 | rs13281615 | 20,645 | 8,312 | 1.13 | 1.09 | - | 1.17 |  | 4,178 | 1.06 | 1.01 | - | 1.11 | 0.011 | 0.35 |
| *LSP1* | rs3817198 | 24,538 | 9,428 | 1.07 | 1.03 | - | 1.11 |  | 4,781 | 1.07 | 1.02 | - | 1.12 | 0.90 | 1.00 |
| European populations | |  |  |  |  |  |  |  |  |  |  |  |  |  |  |
| *FGFR2* | rs2981582 | 24,204 | 9,131 | 1.29 | 1.24 | - | 1.33 | 0 | 4,684 | 1.17 | 1.11 | - | 1.22 | 10^-4^ |  |
| *TNRC9* | rs3803662 | 23,173 | 9,041 | 1.23 | 1.18 | - | 1.28 | 0 | 4,652 | 1.17 | 1.11 | - | 1.22 | 0.076 |  |
| *MAP3K1* | rs889312 | 24,208 | 9,134 | 1.14 | 1.09 | - | 1.18 | 0 | 4,700 | 1.12 | 1.07 | - | 1.18 | 0.66 |  |
| 8q24 | rs13281615 | 20,261 | 8,010 | 1.13 | 1.09 | - | 1.18 | 0 | 4,078 | 1.06 | 1.01 | - | 1.11 | 0.009 |  |
| *LSP1* | rs3817198 | 24,160 | 9,133 | 1.07 | 1.03 | - | 1.12 | 0 | 4,683 | 1.07 | 1.01 | - | 1.12 | 0.78 |  |
| Asian populations | |  |  |  |  |  |  |  |  |  |  |  |  |  |  |
| *FGFR2* | rs2981582 | 383 | 297 | 1.34 | 1.06 | - | 1.70 | 0 | 99 | 0.79 | 0.54 | - | 1.15 | 0.006 |  |
| *TNRC9* | rs3803662 | 365 | 296 | 1.06 | 0.86 | - | 1.32 | 0 | 100 | 1.33 | 0.97 | - | 1.82 | 0.18 |  |
| *MAP3K1* | rs889312 | 382 | 301 | 1.07 | 0.87 | - | 1.33 | 0 | 98 | 1.08 | 0.78 | - | 1.48 | 0.99 |  |
| 8q24 | rs13281615 | 384 | 302 | 1.06 | 0.85 | - | 1.32 | 0 | 100 | 1.07 | 0.78 | - | 1.48 | 0.95 |  |
| *LSP1* | rs3817198 | 378 | 295 | 0.80 | 0.59 | - | 1.09 | 0 | 98 | 1.10 | 0.72 | - | 1.68 | 0.16 |  |

*Adjusted for study. Allele changes are (common>rare based on frequencies in European populations): G>A for rs2981582; G>A for rs3803662; T>G for rs889312; A>G for rs13281615 and A>G for rs3817198.

**P value for heterogeneity calculated from case-only analyses adjusted for study.

***Permutation adjusted P value for heterogeneity.
