# Supplementary material for: Heterogeneity of Breast Cancer Associations with Five Susceptibility Loci by Clinical and Pathological Characteristics
Source: PLoS Genet. 2008 Apr 25;4(4):e1000054. doi: 10.1371/journal.pgen.1000054 (PMC2291027; doi:10.1371/journal.pgen.1000054)
Supplement: Table S8 — Per-allele odds ratios for breast cancer risk by grade, stratified by ethnicity. (0.07 MB DOC) [file pgen.1000054.s011.doc]

Table S8. Per-allele odds ratios for breast cancer risk by grade, stratified by ethnicity

|  |  |  | Grade 1 | | | | |  | Grade 2 | | | | |  | Grade 3 | | | | | Obs. | Adj. |
| --- | --- | --- | --- | --- | --- | --- | --- | --- | --- | --- | --- | --- | --- | --- | --- | --- | --- | --- | --- | --- | --- |
| Locus | SNP | Controls | N | OR* | 95% CI | | |  | N | OR* | 95% CI | | |  | N | OR | 95% CI | | | P** | P*** |
| All populations | |  |  |  |  |  |  |  |  |  |  |  |  |  |  |  |  |  |  |  |  |
| *FGFR2* | rs2981582 | 23,293 | 3,302 | 1.35 | 1.28 | - | 1.42 |  | 7,768 | 1.30 | 1.25 | - | 1.35 |  | 4,883 | 1.14 | 1.09 | - | 1.19 | 10^-8^ | <0.001 |
| *TNRC9* | rs3803662 | 22,291 | 3,273 | 1.25 | 1.19 | - | 1.33 |  | 7,680 | 1.22 | 1.18 | - | 1.27 |  | 4,831 | 1.16 | 1.10 | - | 1.21 | 0.02 | 0.50 |
| *MAP3K1* | rs889312 | 23,301 | 3,288 | 1.10 | 1.04 | - | 1.17 |  | 7,786 | 1.12 | 1.07 | - | 1.16 |  | 4,904 | 1.09 | 1.04 | - | 1.15 | 0.91 | 1.00 |
| 8q24 | rs13281615 | 19,314 | 2,989 | 1.18 | 1.12 | - | 1.25 |  | 6,758 | 1.16 | 1.11 | - | 1.20 |  | 4,351 | 1.06 | 1.01 | - | 1.11 | 10^-4^ | 0.016 |
| *LSP1* | rs3817198 | 23,262 | 3,291 | 1.10 | 1.04 | - | 1.16 |  | 7,775 | 1.08 | 1.04 | - | 1.12 |  | 4,908 | 1.08 | 1.03 | - | 1.14 | 0.77 | 1.00 |
| European populations | |  |  |  |  |  |  |  |  |  |  |  |  |  |  |  |  |  |  |  |  |
| *FGFR2* | rs2981582 | 23,290 | 3,301 | 1.35 | 1.28 | - | 1.42 |  | 7,767 | 1.30 | 1.25 | - | 1.35 |  | 4,880 | 1.14 | 1.09 | - | 1.19 | 10^-8^ |  |
| *TNRC9* | rs3803662 | 22,288 | 3,272 | 1.25 | 1.19 | - | 1.33 |  | 7,679 | 1.22 | 1.18 | - | 1.27 |  | 4,828 | 1.15 | 1.10 | - | 1.21 | 0.02 |  |
| *MAP3K1* | rs889312 | 23,298 | 3,287 | 1.10 | 1.04 | - | 1.17 |  | 7,785 | 1.12 | 1.07 | - | 1.16 |  | 4,901 | 1.09 | 1.04 | - | 1.15 | 0.92 |  |
| 8q24 | rs13281615 | 19,311 | 2,988 | 1.18 | 1.12 | - | 1.25 |  | 6,757 | 1.16 | 1.11 | - | 1.20 |  | 4,348 | 1.06 | 1.01 | - | 1.11 | 10^-4^ |  |
| *LSP1* | rs3817198 | 23,259 | 3,290 | 1.10 | 1.04 | - | 1.16 |  | 7,774 | 1.08 | 1.04 | - | 1.12 |  | 4,905 | 1.08 | 1.03 | - | 1.14 | 0.76 |  |

*Adjusted for study. Allele changes are (common>rare based on frequencies in European populations): G>A for rs2981582; G>A for rs3803662; T>G for rs889312; A>G for rs13281615 and A>G for rs3817198.

** P value for heterogeneity of ORs from case-only analyses adjusted for study, assuming a linear trend with increasing tumor grade.

***Permutation adjusted P value for heterogeneity.

Data for Asian populations is not shown because of very small numbers of Asians with information on tumor grade.
