# Supplementary material for: Heterogeneity of Breast Cancer Associations with Five Susceptibility Loci by Clinical and Pathological Characteristics
Source: PLoS Genet. 2008 Apr 25;4(4):e1000054. doi: 10.1371/journal.pgen.1000054 (PMC2291027; doi:10.1371/journal.pgen.1000054)
Supplement: Table S9 — Per-allele odds ratios for breast cancer risk by nodal status, stratified by ethnicity. (0.07 MB DOC) [file pgen.1000054.s012.doc]

Table S9. Per-allele odds ratios for breast cancer risk by nodal status, stratified by ethnicity

|  |  |  | Cases with negative nodes | | | | |  | Cases with positive nodes | | | | | Observed | Adjusted |
| --- | --- | --- | --- | --- | --- | --- | --- | --- | --- | --- | --- | --- | --- | --- | --- |
| Locus | SNP | Controls | N | OR* | 95% CI | | |  | N | OR* | 95% CI | | | P** | P*** |
| All populations | |  |  |  |  |  |  |  |  |  |  |  |  |  |  |
| *FGFR2* | rs2981582 | 19,261 | 9,304 | 1.25 | 1.20 | - | 1.29 |  | 5,540 | 1.33 | 1.27 | - | 1.39 | 0.013 | 0.41 |
| *TNRC9* | rs3803662 | 18,493 | 9,192 | 1.19 | 1.14 | - | 1.24 |  | 5,500 | 1.20 | 1.14 | - | 1.26 | 0.78 | 1.00 |
| *MAP3K1* | rs889312 | 19,276 | 9,330 | 1.11 | 1.06 | - | 1.15 |  | 5,570 | 1.14 | 1.08 | - | 1.19 | 0.31 | 1.00 |
| 8q24 | rs13281615 | 15,314 | 8,163 | 1.11 | 1.07 | - | 1.15 |  | 4,967 | 1.10 | 1.05 | - | 1.16 | 0.95 | 1.00 |
| *LSP1* | rs3817198 | 19,254 | 9,314 | 1.05 | 1.01 | - | 1.09 |  | 5,560 | 1.08 | 1.03 | - | 1.13 | 0.38 | 1.00 |
| European populations | | |  |  |  |  |  |  |  |  |  |  |  |  |  |
| *FGFR2* | rs2981582 | 18,517 | 8,855 | 1.24 | 1.20 | - | 1.29 |  | 5,226 | 1.32 | 1.27 | - | 1.39 | 0.01 |  |
| *TNRC9* | rs3803662 | 17,766 | 8,743 | 1.19 | 1.14 | - | 1.24 |  | 5,186 | 1.20 | 1.15 | - | 1.27 | 0.63 |  |
| *MAP3K1* | rs889312 | 18,526 | 8,877 | 1.11 | 1.06 | - | 1.15 |  | 5,256 | 1.14 | 1.08 | - | 1.19 | 0.29 |  |
| 8q24 | rs13281615 | 14,563 | 7,708 | 1.11 | 1.07 | - | 1.16 |  | 4,652 | 1.12 | 1.06 | - | 1.17 | 0.89 |  |
| *LSP1* | rs3817198 | 18,508 | 8,865 | 1.05 | 1.01 | - | 1.09 |  | 5,248 | 1.07 | 1.02 | - | 1.13 | 0.55 |  |
| Asian populations | |  |  |  |  |  |  |  |  |  |  |  |  |  |  |
| *FGFR2* | rs2981582 | 744 | 449 | 1.27 | 1.06 | - | 1.53 |  | 314 | 1.38 | 1.13 | - | 1.68 | 0.79 |  |
| *TNRC9* | rs3803662 | 727 | 449 | 1.17 | 0.99 | - | 1.39 |  | 314 | 1.09 | 0.90 | - | 1.33 | 0.40 |  |
| *MAP3K1* | rs889312 | 750 | 453 | 1.11 | 0.94 | - | 1.32 |  | 314 | 1.12 | 0.92 | - | 1.35 | 0.99 |  |
| 8q24 | rs13281615 | 751 | 455 | 1.06 | 0.89 | - | 1.25 |  | 315 | 0.93 | 0.77 | - | 1.12 | 0.43 |  |
| *LSP1* | rs3817198 | 746 | 449 | 0.90 | 0.70 | - | 1.14 |  | 312 | 1.28 | 0.99 | - | 1.66 | 0.06 |  |

*Adjusted for study. Allele changes are (common>rare based on frequencies in European populations): G>A for rs2981582; G>A for rs3803662; T>G for rs889312; A>G for rs13281615 and A>G for rs3817198.

** P value for heterogeneity of ORs from case-only analyses adjusting for study.

***Permutation adjusted P value for heterogeneity.
