# Supplementary material for: Heterogeneity of Breast Cancer Associations with Five Susceptibility Loci by Clinical and Pathological Characteristics
Source: PLoS Genet. 2008 Apr 25;4(4):e1000054. doi: 10.1371/journal.pgen.1000054 (PMC2291027; doi:10.1371/journal.pgen.1000054)
Supplement: Table S10 — Per-allele odds ratios for the association between FGFR2 rs2981582 and breast cancer risk by ER and nodal status. (0.03 MB DOC) [file pgen.1000054.s013.doc]

Table S10: Per-allele odds ratios for the association between *FGFR2* rs2981582 and breast cancer risk by ER and nodal status

| ER status | Nodal status | Cases | OR | 95% CI | | |  | P*** |
| --- | --- | --- | --- | --- | --- | --- | --- | --- |
| Positive | Negative | 5,975 | 1.30 | 1.25 | - | 1.36 |  | (ref.) |
|  | Positive | 3,572 | 1.37 | 1.30 | - | 1.45 |  | 0.14 |
| Negative | Negative | 1,614 | 1.05 | 0.97 | - | 1.13 |  | (ref.) |
|  | Positive | 1,213 | 1.18 | 1.09 | - | 1.29 |  | 0.036 |

*The number of controls included in the analyses is 26,058.

**Adjusted for study. Allele changes are (common>rare based on frequencies in European populations): G>A for rs2981582; G>A for rs3803662; T>G for rs889312; A>G for rs13281615 and A>G for rs3817198.

*** P value for heterogeneity of ORs from case-only analyses adjusting for study.
