# Supplementary material for: Heterogeneity of Breast Cancer Associations with Five Susceptibility Loci by Clinical and Pathological Characteristics
Source: PLoS Genet. 2008 Apr 25;4(4):e1000054. doi: 10.1371/journal.pgen.1000054 (PMC2291027; doi:10.1371/journal.pgen.1000054)
Supplement: Table S11 — Per-allele odds ratios for breast cancer risk by tumor size, stratified by ethnicity. (0.09 MB DOC) [file pgen.1000054.s014.doc]

Table S11. Per-allele odds ratios for breast cancer risk by tumor size, stratified by ethnicity

|  |  |  | Size ≤ 1cm | | | | |  | Size >1-≤2cm | | | | |  | Size>2cm | | | | | Obs. | Adj. |
| --- | --- | --- | --- | --- | --- | --- | --- | --- | --- | --- | --- | --- | --- | --- | --- | --- | --- | --- | --- | --- | --- |
| Locus | SNP | Controls | N | OR* | 95% CI | | |  | N | OR* | 95% CI | | |  | N | OR* | 95% CI | | | P** | P*** |
| All populations | |  |  |  |  |  |  |  |  |  |  |  |  |  |  |  |  |  |  |  |  |
| *FGFR2* | rs2981582 | 11,697 | 1,616 | 1.34 | 1.24 | - | 1.45 |  | 3,413 | 1.27 | 1.21 | - | 1.35 |  | 2,977 | 1.25 | 1.18 | - | 1.33 | 0.14 | 1.00 |
| *TNRC9* | rs3803662 | 10,973 | 1,561 | 1.21 | 1.12 | - | 1.32 |  | 3,315 | 1.26 | 1.18 | - | 1.34 |  | 2,898 | 1.16 | 1.09 | - | 1.24 | 0.24 | 1.00 |
| *MAP3K1* | rs889312 | 11,701 | 1,618 | 1.13 | 1.04 | - | 1.22 |  | 3,419 | 1.08 | 1.02 | - | 1.15 |  | 2,988 | 1.11 | 1.04 | - | 1.18 | 0.82 | 1.00 |
| 8q24 | rs13281615 | 10,919 | 1,550 | 1.16 | 1.07 | - | 1.25 |  | 3,294 | 1.13 | 1.07 | - | 1.20 |  | 2,879 | 1.08 | 1.01 | - | 1.14 | 0.084 | 0.96 |
| *LSP1* | rs3817198 | 11,706 | 1,616 | 1.09 | 1.01 | - | 1.18 |  | 3,433 | 1.06 | 1.00 | - | 1.13 |  | 2,993 | 1.09 | 1.02 | - | 1.16 | 0.94 | 1.00 |
| European populations | |  |  |  |  |  |  |  |  |  |  |  |  |  |  |  |  |  |  |  |  |
| *FGFR2* | rs2981582 | 11,336 | 1,526 | 1.33 | 1.23 | - | 1.43 |  | 3,245 | 1.27 | 1.20 | - | 1.35 |  | 2,771 | 1.25 | 1.17 | - | 1.33 | 0.19 |  |
| *TNRC9* | rs3803662 | 10,611 | 1,470 | 1.20 | 1.10 | - | 1.31 |  | 3,147 | 1.26 | 1.18 | - | 1.34 |  | 2,694 | 1.16 | 1.09 | - | 1.24 | 0.32 |  |
| *MAP3K1* | rs889312 | 11,333 | 1,528 | 1.14 | 1.05 | - | 1.24 |  | 3,251 | 1.09 | 1.02 | - | 1.16 |  | 2,780 | 1.08 | 1.02 | - | 1.16 | 0.35 |  |
| 8q24 | rs13281615 | 10,552 | 1,459 | 1.17 | 1.08 | - | 1.26 |  | 3,126 | 1.14 | 1.08 | - | 1.21 |  | 2,672 | 1.09 | 1.02 | - | 1.16 | 0.10 |  |
| *LSP1* | rs3817198 | 11,338 | 1,525 | 1.08 | 1.00 | - | 1.18 |  | 3,265 | 1.06 | 1.00 | - | 1.12 |  | 2,787 | 1.09 | 1.02 | - | 1.16 | 0.82 |  |
| Asian populations | |  |  |  |  |  |  |  |  |  |  |  |  |  |  |  |  |  |  |  |  |
| *FGFR2* | rs2981582 | 361 | 90 | 1.70 | 1.22 | - | 2.37 |  | 168 | 1.35 | 1.03 | - | 1.76 |  | 206 | 1.40 | 1.09 | - | 1.81 | 0.39 |  |
| *TNRC9* | rs3803662 | 362 | 91 | 1.35 | 0.96 | - | 1.89 |  | 168 | 1.27 | 0.97 | - | 1.66 |  | 204 | 1.17 | 0.92 | - | 1.51 | 0.45 |  |
| *MAP3K1* | rs889312 | 368 | 90 | 0.97 | 0.70 | - | 1.34 |  | 168 | 1.01 | 0.78 | - | 1.30 |  | 208 | 1.42 | 1.11 | - | 1.81 | 0.015 |  |
| 8q24 | rs13281615 | 367 | 91 | 0.95 | 0.69 | - | 1.32 |  | 168 | 0.91 | 0.70 | - | 1.18 |  | 207 | 0.88 | 0.69 | - | 1.12 | 0.66 |  |
| *LSP1* | rs3817198 | 368 | 91 | 1.36 | 0.88 | - | 2.11 |  | 168 | 1.16 | 0.80 | - | 1.67 |  | 206 | 1.12 | 0.79 | - | 1.58 | 0.44 |  |

*Adjusted for study. Allele changes are (common>rare based on frequencies in European populations): G>A for rs2981582; G>A for rs3803662; T>G for rs889312; A>G for rs13281615 and A>G for rs3817198.

**P value for heterogeneity of ORs from case-only analyses adjusted for study, assuming a linear trend with increasing tumor size

***Permutation adjusted P value for heterogeneity.
