# Supplementary material for: Heterogeneity of Breast Cancer Associations with Five Susceptibility Loci by Clinical and Pathological Characteristics
Source: PLoS Genet. 2008 Apr 25;4(4):e1000054. doi: 10.1371/journal.pgen.1000054 (PMC2291027; doi:10.1371/journal.pgen.1000054)
Supplement: Table S12 — Per-allele odds ratios for breast cancer risk by histopathogic subtypes, stratified by ethnicity. (0.06 MB DOC) [file pgen.1000054.s015.doc]

Table S12. Per-allele odds ratios for breast cancer risk by histopathogic subtypes, stratified by ethnicity

|  |  |  | Ductal | | | | |  | Lobular | | | | | Observed | Adjusted |
| --- | --- | --- | --- | --- | --- | --- | --- | --- | --- | --- | --- | --- | --- | --- | --- |
| Locus | SNP | Controls | N | OR* | 95% CI | | |  | N | OR* | 95% CI | | | P** | P*** |
| All populations | |  |  |  |  |  |  |  |  |  |  |  |  |  |  |
| *FGFR2* | rs2981582 | 23,426 | 13,634 | 1.25 | 1.21 | - | 1.29 |  | 2,446 | 1.29 | 1.22 | - | 1.37 | 0.21 | 1.00 |
| *TNRC9* | rs3803662 | 22,670 | 13,396 | 1.21 | 1.17 | - | 1.25 |  | 2,408 | 1.24 | 1.16 | - | 1.32 | 0.40 | 1.00 |
| *MAP3K1* | rs889312 | 23,452 | 13,652 | 1.11 | 1.07 | - | 1.14 |  | 2,447 | 1.16 | 1.09 | - | 1.24 | 0.12 | 0.99 |
| 8q24 | rs13281615 | 19,482 | 12,288 | 1.12 | 1.08 | - | 1.15 |  | 2,055 | 1.14 | 1.07 | - | 1.22 | 0.57 | 1.00 |
| *LSP1* | rs3817198 | 23,409 | 13,643 | 1.07 | 1.04 | - | 1.11 |  | 2,448 | 1.07 | 1.01 | - | 1.14 | 0.97 | 1.00 |
| European populations | |  |  |  |  |  |  |  |  |  |  |  |  |  |  |
| *FGFR2* | rs2981582 | 23,062 | 13,200 | 1.25 | 1.21 | - | 1.29 | 0 | 2,443 | 1.29 | 1.21 | - | 1.37 | 0.22 |  |
| *TNRC9* | rs3803662 | 22,305 | 12,962 | 1.21 | 1.17 | - | 1.25 | 0 | 2,405 | 1.24 | 1.16 | - | 1.32 | 0.43 |  |
| *MAP3K1* | rs889312 | 23,081 | 13,216 | 1.11 | 1.07 | - | 1.14 | 0 | 2,444 | 1.16 | 1.09 | - | 1.24 | 0.14 |  |
| 8q24 | rs13281615 | 19,112 | 11,851 | 1.12 | 1.09 | - | 1.16 | 0 | 2,052 | 1.15 | 1.07 | - | 1.22 | 0.58 |  |
| *LSP1* | rs3817198 | 23,038 | 13,208 | 1.07 | 1.04 | - | 1.11 | 0 | 2,445 | 1.07 | 1.00 | - | 1.14 | 0.91 |  |

*Adjusted for study. Allele changes are (common>rare based on frequencies in European populations): G>A for rs2981582; G>A for rs3803662; T>G for rs889312; A>G for rs13281615 and A>G for rs3817198.

**P value for heterogeneity of ORs from case-only analyses adjusted for study

***Permutation adjusted P value for heterogeneity.

Data for Asian populations is not shown because of small numbers of Asians with information on histopathologycal subtypes.
