# Supplementary material for: Heterogeneity of Breast Cancer Associations with Five Susceptibility Loci by Clinical and Pathological Characteristics
Source: PLoS Genet. 2008 Apr 25;4(4):e1000054. doi: 10.1371/journal.pgen.1000054 (PMC2291027; doi:10.1371/journal.pgen.1000054)
Supplement: Table S13 — Per-allele odds ratios for breast cancer risk by stage at diagnosis, stratified by ethnicity. (0.08 MB DOC) [file pgen.1000054.s016.doc]

Table S13: Per-allele odds ratios for breast cancer risk by stage at diagnosis, stratified by ethnicity

|  |  |  | Stage I | | | | |  | Stage II | | | | |  | Stage III/IV | | | | | Obs. | Adj. |
| --- | --- | --- | --- | --- | --- | --- | --- | --- | --- | --- | --- | --- | --- | --- | --- | --- | --- | --- | --- | --- | --- |
| Locus | SNP | Controls | N | OR* | 95% CI | | |  | N | OR* | 95% CI | | |  | N | OR* | 95% CI | | | P** | P*** |
| All populations | |  |  |  |  |  |  |  |  |  |  |  |  |  |  |  |  |  |  |  |  |
| *FGFR2* | rs2981582 | 17,944 | 6,851 | 1.28 | 1.23 | - | 1.33 |  | 5,949 | 1.26 | 1.26 | - | 1.26 |  | 858 | 1.20 | 1.20 | - | 1.20 | 0.27 | 1.00 |
| *TNRC9* | rs3803662 | 17,661 | 6,849 | 1.21 | 1.15 | - | 1.26 |  | 5,972 | 1.21 | 1.15 | - | 1.26 |  | 859 | 1.26 | 1.14 | - | 1.40 | 0.53 | 1.00 |
| *MAP3K1* | rs889312 | 17,977 | 6,863 | 1.13 | 1.08 | - | 1.18 |  | 5,968 | 1.12 | 1.07 | - | 1.18 |  | 861 | 1.19 | 1.07 | - | 1.32 | 0.78 | 1.00 |
| 8q24 | rs13281615 | 14,724 | 6,159 | 1.13 | 1.09 | - | 1.18 |  | 5,294 | 1.11 | 1.06 | - | 1.16 |  | 642 | 1.10 | 0.98 | - | 1.23 | 0.72 | 0.88 |
| *LSP1* | rs3817198 | 17,899 | 6,848 | 1.06 | 1.02 | - | 1.11 |  | 5,971 | 1.05 | 1.00 | - | 1.09 |  | 860 | 1.25 | 1.13 | - | 1.39 | 0.13 | 0.99 |
| European populations | | |  |  |  |  |  |  |  |  |  |  |  |  |  |  |  |  |  |  |  |
| *FGFR2* | rs2981582 | 17,941 | 6,851 | 1.28 | 1.23 | - | 1.33 |  | 5,948 | 1.26 | 1.21 | - | 1.32 |  | 858 | 1.20 | 1.09 | - | 1.33 | 0.27 |  |
| *TNRC9* | rs3803662 | 17,658 | 6,849 | 1.21 | 1.16 | - | 1.26 |  | 5,971 | 1.21 | 1.15 | - | 1.26 |  | 859 | 1.26 | 1.14 | - | 1.40 | 0.53 |  |
| *MAP3K1* | rs889312 | 17,974 | 6,863 | 1.13 | 1.08 | - | 1.18 |  | 5,967 | 1.12 | 1.07 | - | 1.18 |  | 861 | 1.19 | 1.07 | - | 1.33 | 0.79 |  |
| 8q24 | rs13281615 | 14,721 | 6,159 | 1.13 | 1.09 | - | 1.18 |  | 5,293 | 1.11 | 1.06 | - | 1.16 |  | 642 | 1.10 | 0.98 | - | 1.23 | 0.73 |  |
| *LSP1* | rs3817198 | 17,896 | 6,848 | 1.06 | 1.02 | - | 1.11 |  | 5,970 | 1.05 | 1.00 | - | 1.09 |  | 860 | 1.25 | 1.13 | - | 1.39 | 0.12 |  |

*Adjusted for study. Allele changes are (common>rare based on frequencies in European populations): G>A for rs2981582; G>A for rs3803662; T>G for rs889312; A>G for rs13281615 and A>G for rs3817198.

**P value for heterogeneity of ORs from case-only analyses adjusted for study, assuming a linear trend with increasing stage at diagnosis.

***Permutation adjusted P value for heterogeneity.

Data for Asian populations is not shown because of very small numbers of Asians with information on stage at diagnosis.
